# The comparison of diffusion tensor imaging in human hearts between 1.5 T and 3.0 T

**DOI:** 10.1186/s12880-023-00969-9

**Published:** 2023-01-25

**Authors:** Xiaodan Li, Rui Chen, Xi Xu, Zebin Xiao, Xiaoyu Wei, Yuelong Yang, Zhongping Zhang, Zhigang Wu, Yanjie Zhu, Hui Liu

**Affiliations:** 1 Department of Radiology, Guangdong Provincial People’s Hospital (Guangdong Academy of Medical Sciences), Southern Medical University, Guangzhou, Guangdong Province China; 2 School of Medicine, South China University of Technology, Guangzhou, Guangdong Province China; 3 Paul C. Lauterbur Research Centre for Biomedical Imaging, Shenzhen Institute of Advanced Technology, Chinese Academy of Sciences, Shenzhen, China; 4 Shenzhen College of Advanced Technology, University of Chinese Academy of Sciences, Shenzhen, China; 5 Department of Pathology, Guangdong Provincial People’s Hospital (Guangdong Academy of Medical Sciences), Southern Medical University, Guangzhou, Guangdong Province China; 6 Philips Healthcare China, Guangzhou, China; 7 Guangdong Provincial Key Laboratory of Artificial Intelligence in Medical Image Analysis and Application, Guangdong Provincial People’s Hospital (Guangdong Academy of Medical Sciences), Southern Medical University, Guangzhou, China

**Keywords:** Diffusion tensor imaging, Ex-vivo human heart, Cardiovascular magnetic resonance, Comparison

## Abstract

**Background:**

The aim was to compare the diffusion tensor imaging (DTI) indices derived from human hearts between 1.5 T and 3.0 T scanners. Additionally, the reproducibility of DTI indices was assessed between 1.5 T and 3.0 T scanners.

**Methods:**

A total of 18 ex-vivo hearts were derived from patients who underwent heart transplantation. The DTI schemes were performed at 1.5 T and 3.0 T, respectively. Then, the same slices from each ex-vivo heart were selected for image analysis. The student’s t-test or Wilcoxon-rank test was used to compare the statistical differences. The agreement of DTI indices was mainly reported as the interclass correlation coefficient (ICC).

**Results:**

No significant differences (all *P* > 0.05) were found in the DTI indices between 1.5 T and 3.0 T scanners. Interestingly, the ICC of all DTI indices was relatively lower with a low b-value. The reproducibility of the helix angle (HA) was relatively lower when compared to the other DTI indices.

**Conclusion:**

The DTI indices of ex-vivo human hearts between 1.5 T and 3.0 T scanners had no significant differences. The consistency of DTI indices needed caution using a low b-value with different field strengths, and the relatively low reproducibility of HA should be considered.

**Supplementary Information:**

The online version contains supplementary material available at 10.1186/s12880-023-00969-9.

## Background

Recently, cardiovascular magnetic resonance (CMR) diffusion tensor imaging (DTI) has emerged as a promising technique for the determination of myocardial fiber orientation [1, 2] and tissue characterization. DTI could investigate water diffusion within the tissue and derive additional scalar metrics for quantifying structural integrity [3, 4]. Consequently, CMR DTI has become a non-invasive method to illustrate the alterations of cardiac microstructure in patients with dilated cardiomyopathy (DCM), amyloidosis, and hypertrophic cardiomyopathy in recent studies [1, 2, 5–7].

Theoretically, the DTI indices should remain constant at different field strengths [8]. Since DTI is inherently a low-resolution and low-signal-to-resolution technique, the image quality needs to be seriously concerned [9]. DTI is sensitive to the translational motion of water molecules and a small amount of subject motion can lead to a significant signal phase shift or signal loss, which severely affects image quality [9, 10]. In addition, DTI with single-shot echo-planar imaging (EPI) sequence is commonly used. The EPI sequence is particularly sensitive to susceptibility artifacts and magnetic field inhomogeneities due to a long echo train. Moreover, the magnetic field inhomogeneities and related artifacts that increase with rising field strength probably cause the difference of DTI indices in CMR DTI protocols with different field strengths.

With the growing interests of CMR DTI, it has been performed in human hearts with various MRI systems at different field strengths, mainly at 1.5 T [6, 11, 12] or 3.0 T [2, 13]. The comparison between 1.5 T and 3.0 T DTI scanners, however, is an important and essential procedure for adoption in clinical routine [8, 10, 14]. Because DTI indices should not be influenced by the external magnetic field theoretically, the difference of signal-to-noise ratios caused by field strength may affect the DTI indices [8, 15]. If CMR DTI is sought to be clinically useful, the DTI indices should be robust for the external field strength, which may be helpful for the initial diagnosis and monitoring of the therapeutic effects. However, little is known about the reproducibility and consistency of DTI indices in cardiac at different field strengths. Therefore, the purpose of this prospective study was to acquire the CMR DTI data from ex-vivo human hearts, and compare the DTI indices between 1.5 T and 3.0 T scanners. Furthermore, we investigated the impact of different b-values on the DTI indices, and assessed the consistency and reproducibility of these DTI indices.

## Methods

### Study population

A total of 18 ex-vivo hearts were derived from patients who had undergone heart transplantation due to various cardiovascular causes in Guangdong Provincial People's Hospital from April 2020 to January 2021. This study was approved by the Institutional Review Board of Guangdong Provincial People's Hospital (reference number: KY2020-039–01-01).

### Ex-vivo human heart preparation

The ex-vivo hearts from patients were collected and fixed in 10% buffered formalin within 1 h of excision from the recipients at the time of heart transplantation. All ex-vivo hearts were fixed in 10% buffered formalin for at least 24 h before the CMR examination, and the average time between fixation and CMR examination was three days. It has been reported that CMR DTI indices remained constant 24 h after the initial cross-linking by formalin fixation for several weeks [16].

### Image acquisition

The DTI sequence was scanned at a 3.0 T scanner (Ingenia, Philips Healthcare, Amsterdam, The Netherlands) and a 1.5 T scanner (Achieva, Philips Healthcare, Amsterdam, The Netherlands), respectively. A sixteen-channel receiving array coil was used for the 3.0 T MRI scanner and an eight-channel receive array coil was used for the 1.5 T MRI scanner. The DTI was performed with a multi-shot spin-echo sequence with an EPI readout. Multi-slice short axis views of the heart were acquired covering the entire left ventricular. And a 4-chamber 3D T1WI scan was performed to localize the ex-vivo hearts, and ensured that imaging planes were placed in short-axis view orientation at apical, mid-ventricular and basal. Five diffusion series with 5 different b-values were performed separately on ex-vivo hearts, including b-values of 200 s/mm^2^, 400 s/mm^2^, 600 s/mm^2^, 800 s/mm^2^ and 1000 s/mm^2^, respectively. The detailed imaging parameters are shown in Table 1.

**Table 1:** Overview of the different protocols evaluated in this study

Sequence	Field strength	TR (ms)	TE (ms)	Voxel (mm^3^)	Slices	b-values (s/mm^2^)	Diffusion Directions
DTI_200_16d	3.0 T	2000	53	2 $\times$ 2 $\times$ 5	5	200	16
DTI_400_16d	3.0 T	2000	61	2 $\times$ 2 $\times$ 5	5	400	16
DTI_600_16d	3.0 T	2000	66	2 $\times$ 2 $\times$ 5	5	600	16
DTI_800_16d	3.0 T	2000	70	2 $\times$ 2 $\times$ 5	5	800	16
DTI_1000_16d	3.0 T	2000	74	2 $\times$ 2 $\times$ 5	5	1000	16
DTI_200_16d	1.5 T	2000	55	2 $\times$ 2 $\times$ 5	5	200	16
DTI_400_16d	1.5 T	2000	63	2 $\times$ 2 $\times$ 5	5	400	16
DTI_600_16d	1.5 T	2000	69	2 $\times$ 2 $\times$ 5	5	600	16
DTI_800_16d	1.5 T	2000	73	2 $\times$ 2 $\times$ 5	5	800	16
DTI_1000_16d	1.5 T	2000	77	2 $\times$ 2 $\times$ 5	5	1000	16

### Image analysis

Diffusion tensors were calculated on a pixel-by-pixel basis from the CMR diffusion images and diagonalized to yield three eigenvalues (L1, L2 and L3, sorted in descending order and commonly referred to as primary, secondary and tertiary diffusivities, respectively) and eigenvectors (the corresponding eigenvectors E1, E2 and E3). The mean diffusivity (MD), fractional anisotropy (FA), helix angle (HA), E2 angle (E2A), HA transmural gradient and transverse angle (TA) were obtained by the diffusion tensor eigensystem, and E2A values were stated as absolute from 0 to 90 degrees [17–19]. The HA transmural gradient was calculated by dividing the myocardium into five transmural concentric rings. The mean HA for each ring against the transmural depth was obtained, and the gradient was defined as the slope extracted from the linear regression of the mean HA [19].

**Fig. 1:**
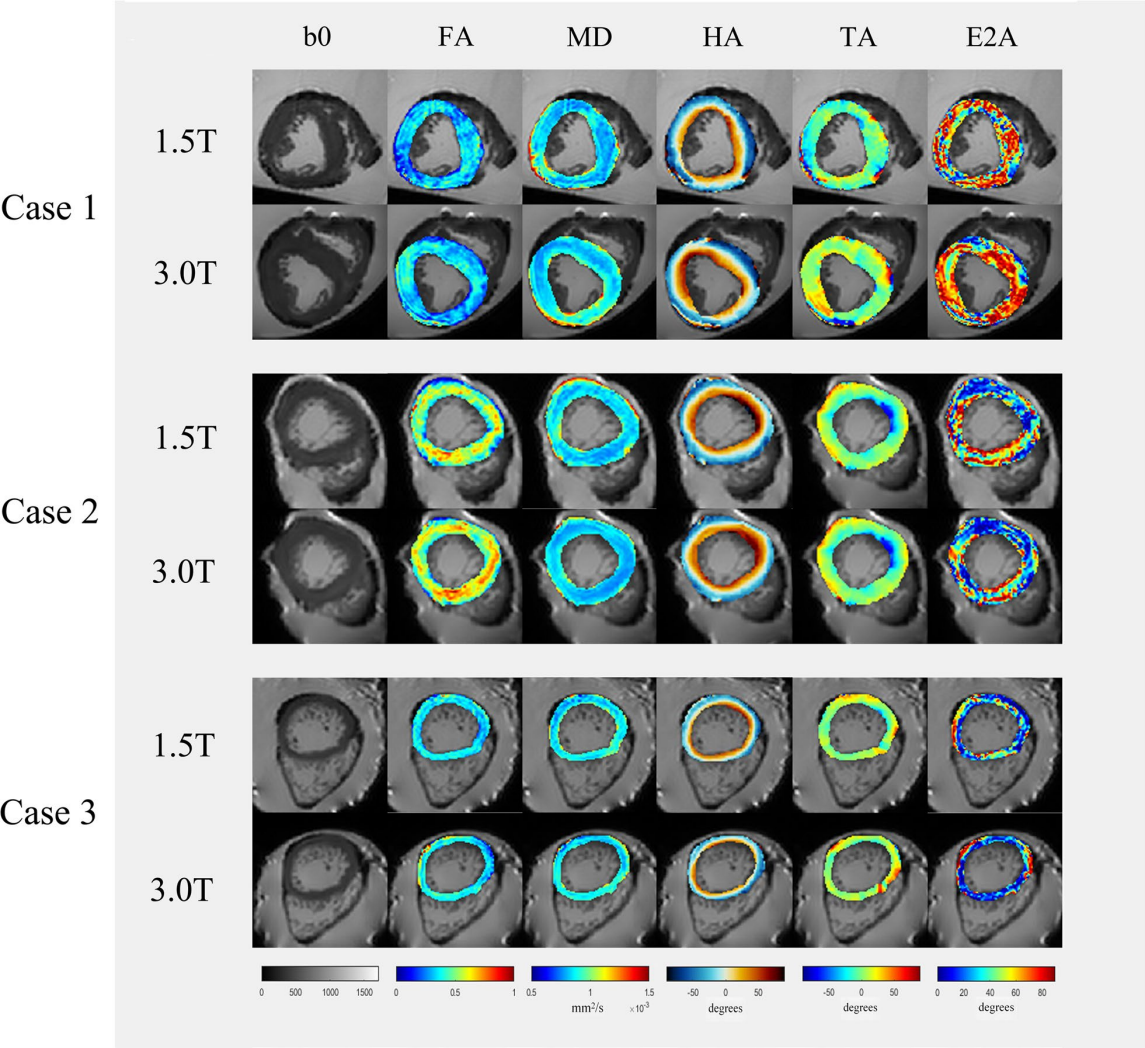
The region of interests of DTI images and DTI indices analysis. FA, fractional anisotropy; MD, mean diffusivity; HA, helix angle; E2A, E2 angle; TA, transverse angle

In the present study, we selected only one slice without any myocardium excision for the image analysis of each ex-vivo heart. All the DTI indices were reported as the average values over the entire left ventricular myocardial area on the selected slice for each ex-vivo heart. All the post-processing computation was performed as Fig. 1 using custom codes written in Matlab (Version R2019a, Mathworks, Natick, MA, USA).

### Statistical analysis

Continuous variables were described as mean ± standard deviation. The normal distribution of variables was tested using the Shapiro–Wilk test. The Student’s t-test was used to compare the means of normally distributed variables, while the Wilcoxon-rank test was used to compare the median of skewed variables. The agreement of DTI indices between 1.5 T and 3.0 T scanners was tested by calculating mean bias and 95% limits of agreement on a Bland–Altman test, and interclass correlation coefficient (ICC).

For the reproducibility analysis, we randomly selected one slice from each ex-vivo heart and sketched the region of interest (ROI) on the left ventricular myocardium. To assess intra-observer reproducibility, the first experienced observer was blinded to the clinical information, and then sketched ROIs twice at 2-week intervals. For inter-observer reproducibility, another experienced observer did the same thing in the selected images. The agreement of reproducibility was also tested by calculating mean bias and 95% limits of agreement on a Bland–Altman test, and ICC. All tests were two-sided, and *P* values < 0.05 were considered statistically significant.

All statistical analyses were performed with the statistical software GraphPad Prism (version 6.0; GraphPad Software, San Diego, California, USA) and in R version 3.4.1 (R Foundation, Vienna, Austria).

## Results

### Study Population

A total of 18 patients (41 ± 17 years, 11 males) were included in the analysis who had undergone heart transplantation. In detail, nine patients had dilated cardiomyopathy, five had coronary heart disease, one had rheumatic heart disease, one had Arrhythmia right ventricular cardiomyopathy, one had cardiac malignancy, and one had myocarditis proven by pathology.

### The comparison between 1.5 T and 3.0 T

Among the DTI indices analyzed, no significant differences (all *P* > 0.05) were found between 1.5 T and 3 T scanners (Table 2). Interestingly, the ICCs of all DTI indices between 1.5 T and 3.0 T scanners were relatively low with a low b-value. In detail, the ICC of FA, MD, HA, E2A, HA transmural gradient, and TA between 1.5 T and 3.0 T were 0.395 (− 0.074, 0.721), 0.319 (− 0.160, 0.676), 0.726 (0.403, 0.888), 0.556 (0.134, 0.807), 0.024 (− 0.437, 0.474), and 0.107 (− 0.367, 0.536), respectively, when the b-value was 200 s/mm^2^. The ICC of FA, MD, HA, E2A, HA transmural gradient, TA between 1.5 T and 3.0 T were 0.903 (0.760, 0.963), 0.937 (0.841, 0.976), 0.957 (0.889, 0.984), 0.835 (0.613, 0.935), 0.400 (− 0.067, 0.724), and 0.306 (− 0.174, 0.668), respectively, when the b-value equaled 1000 s/mm^2^ (Table 3). The mean absolute difference and 95% confidence interval of the mean difference of measurements between 1.5 T and 3.0 T were shown in Table 4 and Additional file 1: Figure S1.

**Table 2:** Overall comparison of DTI indices between 1.5 T and 3.0 T

b-value (s/mm^2^)	Field strength	Fractional anisotropy	Mean diffusivity (mm^2^/s)	Helix angle (degrees)	E2 angle (degrees)	Helix angle transmural gradient (degrees/%trans)	Transverse angle (degrees)
200	1.5 T	0.343 ± 0.054	0.00077 ± 0.00022	− 1.332 ± 8.06	36.45 ± 7.08	− 0.256 ± 0.16	− 0.347 ± 3.44
	3.0 T	0.324 ± 0.088	0.00078 ± 0.00015	0.616 ± 7.80	34.86 ± 8.26	− 0.319 ± 0.19	1.419 ± 5.51
400	1.5 T	0.312 ± 0.055	0.00074 ± 0.00011	− 1.837 ± 8.75	35.09 ± 8.05	− 0.308 ± 0.13	− 0.358 ± 4.25
	3.0 T	0.287 ± 0.068	0.00074 ± 0.00014	0.009 ± 8.19	33.98 ± 10.45	− 0.329 ± 0.18	− 0.051 ± 5.14
600	1.5 T	0.299 ± 0.057	0.00069 ± 0.00010	− 1.110 ± 8.10	34.19 ± 9.85	− 0.331 ± 0.14	− 0.394 ± 3.77
	3.0 T	0.278 ± 0.063	0.00072 ± 0.00014	0.751 ± 8.37	33.74 ± 11.55	− 0.334 ± 0.21	− 0.624 ± 5.26
800	1.5 T	0.296 ± 0.059	0.00067 ± 0.00011	− 1.004 ± 9.11	34.57 ± 10.44	− 0.341 ± 0.13	− 0.662 ± 3.89
	3.0 T	0.273 ± 0.060	0.00070 ± 0.00014	0.630 ± 8.71	33.594 ± 11.64	− 0.348 ± 0.19	− 0.726 ± 4.87
1000	1.5 T	0.286 ± 0.049	0.00066 ± 0.00012	− 0.080 ± 9.98	33.641 ± 10.82	− 0.352 ± 0.17	− 1.201 ± 4.41
	3.0 T	0.273 ± 0.057	0.00067 ± 0.00013	1.021 ± 9.31	32.24 ± 11.71	− 0.350 ± 0.20	− 0.749 ± 5.18

*indicated the significant difference between 1.5 T and 3.0 T. Values in bold indicate significance

The E2A values were stated as absolute from 0 to 90 degrees

**Table 3:** intraclass correlation coefficients of DTI indices between 1.5 T and 3.0 T

b-value	Fractional anisotropy	Mean diffusivity	Helix angle	E2 angle	Helix angle transmural gradient	Transverse angle
200	0.395 (− 0.074, 0.721)	0.319 (− 0.160, 0.676)	0.726 (0.403, 0.888)	0.556 (0.134, 0.807)	0.024 (− 0.437, 0.474)	0.107 (− 0.367, 0.536)
400	0.575 (0.162, 0.817)	0.715 (0.385, 0.883)	0.867 (0.680, 0.948)	0.743 (0.434, 0.895)	0.178 (− 0.302, 0.586)	0.303 (− 0.177, 0.667)
600	0.653 (0.281, 0.854)	0.887 (0.723, 0.956)	0.910 (0.777, 0.965)	0.758 (0.462, 0.902)	0.493 (0.048, 0.774)	0.415 (− 0.050, 0.732)
800	0.679 (0.324, 0.867)	0.916 (0.790, 0.968)	0.954 (0.881, 0.982)	0.821 (0.584, 0.929)	0.285 (− 0.196, 0.655)	0.368 (− 0.105, 0.705)
1000	0.903 (0.760, 0.963)	0.937 (0.841, 0.976)	0.957 (0.889, 0.984)	0.835 (0.613, 0.935)	0.400 (− 0.067, 0.724)	0.306 (− 0.174, 0.668)

“()” indicated the 95% coincident interval of intraclass correlation coefficients

**Table 4:** Mean absolute difference of DTI indices between 1.5 T and 3.0 T

b-value (s/mm^2^)	Fractional anisotropy	Mean diffusivity (mm^2^/s)	Helix angle(degrees)	E2 angle (degrees)
200	− 0.020 (− 0.178, 0.137)	− 0.00001 (− 0.00042, 0.00044)	1.95 (− 9.57, 13.46)	− 1.60 (− 15.80, 12.61)
400	− 0.025 (− 0.137, 0.087)	0.000005 (− 0.00018, 0.00019)	1.93 (− 6.63, 10.49)	− 1.17 (− 14.23, 12.00)
600	− 0.023 (− 0.120, 0.075)	− 0.00003 (− 0.00009, 0.00014)	1.86 (− 4.98, 8.71)	− 0.45 (− 15.08, 14.17)
800	− 0.023 (− 0.117, 0.071)	− 0.00002 (− 0.00008, 0.00012)	1.63 (− 3.69, 6.95)	− 0.98 (− 13.94, 11.98)
1000	− 0.013 (− 0.060, 0.033)	− 0.00001 (− 0.00007, 0.00010)	1.10 (− 4.44, 6.65)	− 0.41 (− 13.09, 12.27)

b-value (s/mm^2^)	Transverse angle (degrees)	Helix angle transmural gradient (degrees/%trans)
200	1.77 (− 11.64, 15.17)	− 0.063 (− 0.538, 0.411)
400	0.31 (− 10.61, 11.22)	− 0.021 (− 0.412, 0.370)
600	− 0.23 (− 9.93, 9.47)	− 0.003 (− 0.354, 0.348)
800	− 0.065 (− 9.78, 9.66)	− 0.007 (− 0.385, 0.371)
1000	0.46 (− 10.64, 11.57)	0.002 (− 0.388, 0.392)

“()” indicated the 95% coincident interval of intraclass correlation coefficients

### The difference among various b-values

**Fig. 2:**
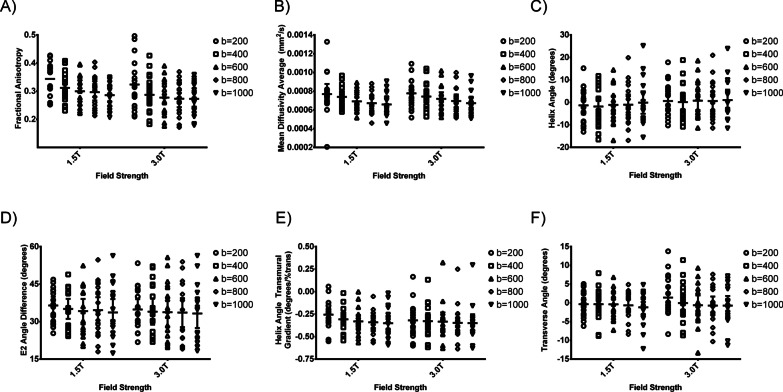
The bar chart shows DTI indices with different b-values. **A** fractional anisotropy; **B** mean diffusivity; **C** helix angle; **D** E2 angle; **E** helix angle transmural gradient; **F** transverse angle. The E2A values were stated as absolute from 0 to 90 degrees

As the b-value increased, MD decreased at both 1.5 T and 3.0 T scanners, while FA, HA, E2A, HA transmural gradient, and TA did not show significant trend varied with b-values, which were shown in Fig. 2. In Fig. 2B, MD with a b-value of 200 s/mm^2^ was significantly higher than MD with a b-value of 1000 s/mm^2^ (*P* = 0.032) at 3.0 T. In addition, HA, E2A, HA transmural gradient and TA at both 1.5 T and 3.0 T did not show any statistical difference with different b-values.

### Reproducibility

The intra- and inter-observer reproducibility of DTI indices were shown in Table 5 and Additional file 1: Figure S2. The intra-observer ICC and inter-observer ICC of FA, MD, E2A, HA transmural gradient, and TA were all over 0.85. However, the intra-observer ICC and inter-observer ICC of HA were the lowest. The intra-observer ICC of HA was 0.888 (0.726, 0.957), while the inter-observer ICC was 0.814 (0.569, 0.926).

**Table 5:** Intraclass correlation coefficients (ICC) and mean absolute difference of DTI indices measurement

DTI indices	Intra-observer ICC	Intra-observer Bias (limits of agreement)	Inter-observer ICC	Inter-observer Bias (limits of agreement)
Fractional anisotropy	0.984 (0.957, 0.994)	− 0.0030 (− 0.018, 0.024)	0.970 (0.922, 0.989)	0.0023 (− 0.018, 0.023)
Mean diffusivity	0.973 (0.929, 0.990)	− 0.000020 (− 0.000061, 0.000021)	0.980 (0.948, 0.993)	0.0000007 (− 0.000026, 0.000027)
Helix angle	0.888 (0.726, 0.957)	2.21 (− 5.48, 9.91)	0.814 (0.569, 0.926)	1.44 (− 8.00, 10.87)
E2 angle	0.969 (0.920, 0.988)	− 2.17 (− 6.52, 2.18)	0.939 (0.845, 0.977)	− 3.18 (− 8.82, 2.47)
Helix angle transmural gradient	0.922 (0.805, 0.970)	0.021 (− 0.124, 0.166)	0.887 (0.724, 0.956)	0.030 (− 0.149, 0.210)
Transverse angle	0.934 (0.834, 0.975)	0.688 (− 2.98, 4.35)	0.892 (0.735, 0.958)	1.379 (− 3.57, 6.33)

“()” indicated the 95% coincident interval of intraclass correlation coefficients or mean difference using Bland–Altman test

## Discussion

The DTI has emerged as a promising technique in CMR, which was mainly performed with human hearts at 1.5 T or 3.0 T scanners. Given present trends, the DTI scanning in ex-vivo human hearts was performed, and the DTI indices and reproducibility were compared between 1.5 T and 3.0 T scanners. The main findings of our study were included as follows: (1) There were no significant differences in DTI indices between 1.5 T and 3.0 T; (2) the ICC of all DTI indices between 1.5 T and 3.0 T was relatively low with a low b-value; (3) the reproducibility of HA was relatively lower compared with the other DTI indices.

Theoretically, DTI indices should not be influenced by the external magnetic field. However, the comparison of DTI indices at 1.5 T and 3.0 T scanners seemed to reveal contradicting findings in clinical practice. In the evaluation of the brain, Hunsche et al. concluded that MD and FA did not differ significantly comparing 1.5 T and 3.0 T [14], while Guilfoyle et al. observed the changes of DTI indices varied with field length [20]. In the evaluation of kidneys, Notohamiprodjo et al. concluded that none of the DTI indices did not differ significantly when comparing 1.5 T and 3.0 T, while Kido et al. [10] found that both FA and MD were significantly different at different field strengths. The other reason might be the different settings with Lohr et al. [21] reported that there is no difference found at porcine hearts between CMR DTI at 3.0 T and 7.0 T, similar to the present study. However, the comparison of field strength was different between Lohr’s and our study (3.0 T/7.0 T vs. 1.5 T/ 3.0 T). Furthermore, the human ex-vivo hearts were fixed in Formalin in this study, while the porcine hearts were not fixed in Formalin or other substances in Lohr’s study [21]. Whether the DTI indices in the specimens without fixation varied with time was still unclear, and whether it affected their findings remained unknown. Further experiments are urgently needed before the CMR DTI technique is used in clinical practice.

The difference of DTI indices varied with b-value in CMR DTI has not been reported yet, and the b-value mostly ranged from 200 s/mm^2^ to 1000 s/mm^2^ in recent research [2, 6, 17, 22, 23]. In the present study, DTI indices derived from the 1.5 T and 3.0 T with 5 different b-values were analyzed, respectively. It was shown that the MD was affected by b-value in the present study, which was similar to Chou et al. They reported that DTI indices were influenced by echo time and b-value, the variations of MD slightly increased with echo time but decreased with the b-value [15]. The reproducibility and accuracy of DTI indices were both improved by increasing the b-value, when the b-value was lower than 1000 s/mm^2^. In the present study, the ICC of FA and MD was the highest when the b-value equaled 1000 s/mm^2^, which was similar to the results of Chou et al.

The reproducibility of the DTI indices was also essential, in line with other similar studies in different organs [8, 24]. In the present study, we randomly selected one slice from each ex-vivo heart and sketched ROIs of the myocardium. Not surprisingly, it was shown that the ICC of FA and MD were all over 0.95 in CMR DTI, because FA and MD were the most widely used diffusion scalars in DTI. The reproducibility of HA has not been reported yet. In the present study, the ICC of HA was lower than the other diffusion scalars. The intra-observer ICC of HA was 0.888 (0.726, 0.957), while the inter-observer ICC was 0.814 (0.569, 0.926). Though some of the studies showed good reproducibility of HA in normal control and in patients with hypertrophic cardiomyopathy [25, 26], however, in the present study, half of the patients who underwent heart transplantation were diagnosed DCM. In patients with DCM, the thickness of the myocardium was commonly decreased in the end stage, and the thin myocardium would probably cause the value of HA more sensitive to the sketched ROIs. That might have it challenged the accurate quantification of HA, resulting in the relatively lower ICC of HA [5].

### Limitations

There were several limitations in the present study when interpreting the results. First, the sample sizes of the study need to be improved, and the results should be also proved using the MRI systems from other manufacturers as well in the future. Second, the excision of the ex-vivo hearts could be found in some of the slices, but we only analyzed the slices without myocardium excision to address this problem. Third, the ex-vivo hearts were fixed by formalin, and the DTI indices could be different from the ones from in-vivo hearts. However, the present study focused on comparing the DTI indices between 1.5 T and 3.0 T scanners, given the same ex-vivo hearts. At last, the inconsistent echo time and large slice thickness might be the potential limitations in the present study.

## Conclusion

In conclusion, the magnetic resonance DTI indices of ex-vivo human hearts between 1.5 T and 3.0 T showed no significant difference in the present study. However, the consistency of DTI indices needed caution using a low b-value with different field strengths, and the relatively low reproducibility of HA should be considered in clinical research or clinical practice in the future using the CMR DTI technique.

## Supplementary Information

**Additional file 1**. Supplementary figures.

## Data Availability

The datasets generated and analyzed during the current study are not publicly available due to the data being also a part of an ongoing study but are available from the corresponding author on reasonable request.

## Abbreviations

CMR: Cardiovascular magnetic resonance
DTI: Diffusion tensor imaging
EPI: Echo-planar imaging
DCM: Dilated cardiomyopathy
FA: Fractional anisotropy
MD: Mean diffusivity
HA: Helix angle
E2A: E2 angle
TA: Transverse angle
ICC: Interclass correlation coefficient
ROI: Region of interest
