# Supplementary material for: The comparison of diffusion tensor imaging in human hearts between 1.5 T and 3.0 T
Source: BMC Med Imaging. 2023 Jan 25;23:14. doi: 10.1186/s12880-023-00969-9 (PMC9875455; doi:10.1186/s12880-023-00969-9)
Supplement: Supplementary file 1 — Additional file 1. Supplementary figures. [file 12880_2023_969_MOESM1_ESM.docx]

**List of Figures**

**Figure S1**

The Bland–Altman plots showed the difference of DTI indices between 1.5T and 3.0T

**Figure S1_1.** The Bland–Altman plots showed the difference of DTI indices between 1.5T and 3.0T with b-value of 200 s/mm^2^

**Figure S1_2.** The Bland–Altman plots showed the difference of DTI indices between 1.5T and 3.0T with b-value of 400 s/mm^2^

**Figure S1_3.** The Bland–Altman plots showed the difference of DTI indices between 1.5T and 3.0T with b-value of 600 s/mm^2^.

**Figure S1_4.** The Bland–Altman plots showed the difference of DTI indices between 1.5T and 3.0T with b-value of 800 s/mm^2^.

**Figure S1_5.** The Bland–Altman plots showed the difference of DTI indices between 1.5T and 3.0T with b-value of 1000 s/mm^2^.

**Figure S2.**

The Bland–Altman plots showed the reproducibility of DTI indices measurement

**Figure S2_1.** The Bland–Altman plots showed the intra-observer reproducibility of DTI indices measurement.

**Figure S2_2.** The Bland–Altman plots showed the inter-observer reproducibility of DTI indices measurement.





**Figure S1_1.** The Bland–Altman plots showed the difference of DTI indices between 1.5T and 3.0T with b-value of 200 s/mm^2^.

(A) fractional anisotropy; (B) mean diffusivity; (C) helix angle; (D) E2 angle; (E) helix angle transmural gradient; (F) transverse angle





**Figure S1_2.** The Bland–Altman plots showed the difference of DTI indices between 1.5T and 3.0T with b-value of 400 s/mm^2^.

(A) fractional anisotropy; (B) mean diffusivity; (C) helix angle; (D) E2 angle; (E) helix angle transmural gradient; (F) transverse angle





**Figure S1_3.** The Bland–Altman plots showed the difference of DTI indices between 1.5T and 3.0T with b-value of 600 s/mm^2^.

(A) fractional anisotropy; (B) mean diffusivity; (C) helix angle; (D) E2 angle; (E) helix angle transmural gradient; (F) transverse angle





**Figure S1_4.** The Bland–Altman plots showed the difference of DTI indices between 1.5T and 3.0T with b-value of 800 s/mm^2^.

(A) fractional anisotropy; (B) mean diffusivity; (C) helix angle; (D) E2 angle; (E) helix angle transmural gradient; (F) transverse angle





**Figure S1_5.** The Bland–Altman plots showed the difference of DTI indices between 1.5T and 3.0T with b-value of 1000 s/mm^2^.

(A) fractional anisotropy; (B) mean diffusivity; (C) helix angle; (D) E2 angle; (E) helix angle transmural gradient; (F) transverse angle





**Figure S2_1.** The Bland–Altman plots showed the intra-observer reproducibility of DTI indices measurement.

(A) fractional anisotropy; (B) mean diffusivity; (C) helix angle; (D) E2 angle; (E) helix angle transmural gradient; (F) transverse angle





**Figure S2_2.** The Bland–Altman plots showed the inter-observer reproducibility of DTI indices measurement.

(A) fractional anisotropy; (B) mean diffusivity; (C) helix angle; (D) E2 angle; (E) helix angle transmural gradient; (F) transverse angle
